# Forensic utilization of NGS-STRs and evaluation of system efficacy for different kinship identifications

**DOI:** 10.1186/s40246-025-00858-y

**Published:** 2025-12-24

**Authors:** Hui Xu, Hongbing Yao, Xi Yuan, Qiong Lan, Yifeng Lin, Xiaolian Wu, Qinglin Liang, Qinglin Liu, Lisiteng Luo, Bofeng Zhu

**Affiliations:** 1 Guangzhou Key Laboratory of Forensic Multi-Omics for Precision Identification, School of Forensic Medicine, Southern Medical University, Guangzhou, 510515 Guangdong China; 2 Belt and Road Research Center for Forensic Molecular Anthropology, Key Laboratory of Evidence Science of Gansu Province, Gansu University of Political Science and Law, Lanzhou, 730070 China; 3 Microbiome Medicine Center, Department of Laboratory Medicine, Zhujiang Hospital, Southern Medical University, Guangzhou, 510280 Guangdong China; 4 Key Laboratory of Forensic Medicine in Shanxi Province, School of Forensic Medicine, Shanxi Medical University, Jinzhong, 030600 People’s Republic of China

**Keywords:** Short tandem repeat, Next-generation sequencing, Kinship identification, Baoan people

## Abstract

**Background:**

The accurate identification of complex kinship relationships remains a significant challenge in forensic practice. Traditional kinship identification methods, which primarily rely on length polymorphisms of short tandem repeats (STRs), often face difficulty in achieving sufficient discriminatory power for complex relationships due to the limited genetic information they provide. While next-generation sequencing (NGS) enables the detection of allelic sequence polymorphisms within STRs, its practical value for different kinship analyses in specific populations requires comprehensive evaluation. To this end, the present study investigated the genetic polymorphisms of 52 STRs, with a focus on both length and sequence variations, aiming to evaluate the system efficacy and forensic application value of the amplification system in the forensic identification of complex kinship analyses.

**Results:**

The 52 STRs were highly polymorphic in the studied Baoan group. The acquisition of sequence polymorphism information significantly enhanced the genetic polymorphisms of STRs, and the number of alleles increased by 61.00% compared to length-based polymorphisms alone. Kinship performance was evaluated by simulating 1,000 kinship pairs and 1,000 unrelated individual pairs on the basis of the allele frequencies of 52 STRs, and the influence of different combinations of STR loci on the identification efficacies of different kinship was assessed by using the likelihood ratio (LR) method and identical by state (IBS) method, respectively. When the LR was greater than 10,000 or less than 0.0001 as the judgment threshold, the system efficacy of the 52 STR loci based on sequence polymorphisms for forensic identifications of full siblings and unrelated individuals was 99.85%, and those of half-siblings, grandparents-grandchildren, uncle–nephews and unrelated individuals were 61.50%, 60.95%, and 61.00%, respectively.

**Conclusions:**

The availability of sequence polymorphism data and the increased number of STR loci could enhance the efficacy of the detection systems for kinship identifications to a certain extent. These results highlight the potential of combining length and sequence polymorphisms of STRs in forensic practice, offering a valuable tool for addressing the challenge of complex kinship identification. Future research should validate these thresholds in casework samples and expand the number of STR loci to enhance the detection efficacy for distant relationships.

**Supplementary Information:**

The online version contains supplementary material available at 10.1186/s40246-025-00858-y.

## Background

Short tandem repeats (STRs) are the most widely used genetic markers in forensic identification, which are a type of DNA sequence with length polymorphisms and a variable number of repeats in microsatellite DNA [1, 2]. Capillary electrophoresis (CE) has become the primary testing platform for STRs in major forensic laboratories due to its advantages of high sensitivity, simplicity and rapid operation [3, 4]. Currently, autosomal STR detection is mostly performed by commercialized detection panels, which often contain loci designed based on the Combined DNA Index System (CODIS) [5, 6]. The iterative commercialized detection panels have been widely used for first-degree kinship identification due to their high accuracy and simplicity [7, 8]. Despite the STR detection by CE can meet the needs of kinship testing in most conventional cases, its efficacy for distant kinship identification is often less than 50% due to the limited length polymorphism information [9, 10].

Furthermore, STR testing based on the CE platform can only obtain the genotyping of allele length polymorphisms, and the genotyping results cannot differentiate between alleles with the same length but different sequences. For repetitive structures of such STRs, kinship analysis based solely on length polymorphisms may lack sufficient statistical support, which can lead to inconclusive results, particularly given the variability in classification criteria across laboratories [11]. Besides, in CE detection systems, the number of STRs that can be multiplexed is limited primarily by the restricted number of fluorescent dye channels available for detection and the necessity for size separation resolution, which requires amplicon length differentiations. Although the amplicon lengths of STR loci are determined by primer design and vary across kits, many STR loci in CE kits are intentionally designed with larger size ranges to ensure allelic resolution [12]. Due to the limited number of genetic markers that can be accommodated by the CE platform, how to acquire genotyping data of more genetic markers at one time and obtain more sufficient evidence is still one of the main challenges faced by current forensic practitioners [13]. In addition, due to the relatively high mutation rate of STRs, genotyping based on the CE platform is burdened by the loss of alleles due to mutations in the primer binding regions and the interference of stutter peaks caused by replication slippage, which has limited the further application of STRs in the identification of complex kinship. Therefore, utilizing CE-based STR genotyping to achieve the identification of complex kinship in the second degree or above remains an existing challenge in forensic genetic research [14].

In recent years, with the emergence of high-throughput sequencing technologies, STR detection based on next-generation sequencing (NGS) platforms has been gradually applied in various research fields of forensic science [15–18]. NGS-based detection technology, also known as massively parallel sequencing (MPS), is mainly characterized by high throughput and short read lengths. It can obtain sequence genotyping information of STRs, which to some extent makes up for the limitation of CE typing, substantially improving allele polymorphisms and the identification effectiveness of the detection system [19]. An additional challenge lies in the individual identification of some aged and degraded samples, where DNA has been highly fragmented due to the influence of environmental and other factors. Thus, platforms relying on long amplicons, such as CE-based systems, are prone to the loss of alleles and ineffective amplification, as fragmented DNA struggles to support the amplification of long target sequences [20]. NGS technology overcomes this limitation by enabling the design of shorter amplicons, allowing detection even in samples with DNA fragments below 100 bp [21]. Furthermore, sequence polymorphism information that is lost by the CE platform can be retained using NGS technology, so that the combined information on both length polymorphisms and sequence polymorphisms of the STR alleles can provide stronger evidence for individual identification [22–25]. In the identification of complex kinship relationships, NGS technology allows for the simultaneous acquisition of sequence information in multiple genetic markers; the combination application of multiple genetic markers can help to improve system performance in the identification of complex kinship [26, 27]. Taking the potential application of NGS technology as a basis in the forensic field, different scholars have successively developed a series of multiplex amplification systems and attempted to increase the number of STR loci in the constructed detection system to enhance forensic performance [28–33]. However, most kinship analyses based on NGS-STR have focused on some large populations, with scarce data available for ethnic minorities, and their performance remains unquantified in many unique ethnic groups like the Baoan group [34].

According to the 7th national census, the Baoan group, one of the smallest officially recognized ethnic minorities in China, has a population of approximately 24,000 individuals. They mainly inhabit the Jishishan Baoan, Dongxiang and Salar Autonomous County in Gansu Province. The origins of the Baoan people still remain subject to academic disputes. The prevailing view holds that they emerged from the ethnic integration in the northwestern border areas since the Yuan Dynasty. Their language, the Baoan dialect, belongs to the Mongolic branch of the Altaic language family. However, due to the long-term interaction with the surrounding Han population and the Hui group, a large number of Chinese loanwords have been incorporated into the Baoan dialect, and Chinese has become the primary tool for written communication [35].

Previous STR research on the Baoan group have been limited to 15 CE-STR loci, lacking sequence polymorphism information and high-resolution data necessary for complex kinship analysis [36]. Moreover, a systematic assessment of the extent to which sequence polymorphisms improve the discriminations of various second- and third-degree relationships within a specific group is essential to translate the theoretical advantages of NGS into practical forensic applications [37]. Therefore, this study aims to fill these gaps by investigating the genetic polymorphisms of 52 STRs in the Baoan people based on an NGS panel [31], which incorporated 20 CODIS STRs and 32 non-CODIS STRs, making a total of 52 autosomal STRs. In the present study, the forensic parameters of these 20 CODIS STRs and 32 non-CODIS STRs were evaluated through two dimensions: length polymorphisms and sequence polymorphisms, respectively. In the meantime, the system efficacy of these CODIS and non-CODIS STRs were evaluated in the identifications of different kinship within the third degree using the likelihood ratio (LR) method and the identical by state (IBS) method, respectively. The specific objectives were: (1) to establish the first high-resolution reference database of both length and sequence polymorphisms for the Baoan group; (2) to quantitatively evaluate the enhanced efficacy offered by sequence polymorphisms for identifying different degrees of kinship, from parent-child to first-cousin relationships. The findings will provide valuable population genetic resource tailored to understudied ethnic minority and a robust assessment of the NGS-STR system’s utility for complex kinship identifications in forensic practice.

## Methods

### Sample information and ethical declaration

According to the principle of informed consent, whole blood samples were collected from 138 healthy unrelated individuals from the Baoan group in Gansu Province, China. A small portion of each collected whole blood sample was applied to the FTA cards to prepare the bloodstain samples, which were dried at room temperature and stored for experimental purposes. This study was approved by the Medical Ethics Committee of Zhujiang Hospital of Southern Medical University (Approval Number: 2023-KY-097-02), and both the study objectives and the experimental procedures were reviewed. The study followed the ethical principles of the Declaration of Helsinki of the World Medical Association.

### Principles for loci selection

According to Fan et al., when selecting the 32 non-CODIS STR loci, the following criteria were applied to ensure high forensic utility and compatibility with existing systems: (1) forensic informativeness: all selected non-CODIS STRs have been reported to exhibit high levels of genetic polymorphisms, which enhance the discriminatory power for individual identification and system efficacy for complex kinship analysis; (2) compatibility with existing CE systems: the STR loci were chosen to cover a wide range of commonly used commercial CE-based kits, ensuring data comparison and integration between NGS-based genotypes and the existing forensic DNA databases generated by CE technology; (3) rigorous genomic audit: each marker underwent comprehensive genomic validation, including confirmation of amplification specificity, physical positions, core repeat structures, and flanking sequences, ensuring accurate alignment and sequencing-based genotyping [31].

### Library preparation

The bloodstain samples were pretreated by the direct amplification method, and a piece of 1 mm^2^ bloodstain was used as the DNA template. Library construction was performed using the Forensic Analysis System Multiplecues SetB amplification system (DeepReads, Guangzhou, China) [31]. The program setting for the first round of PCR included initial denaturation at 95 °C for 3 min, followed by 16 cycles of denaturation at 95 °C for 20 s, then annealing and extension at 60 °C for 6 min. The final extension was done at 72 °C for 2 min. The PCR products of the initial round were stored at 4 °C and subsequently purified using DNA Clean Beads (Enlighten, Shanghai, China). The programmed setup for the second round of PCR consisted of initial denaturation at 95 °C for 1 min, followed by 14 cycles of denaturation at 95 °C for 20 s, annealing at 63 °C for 20 s, then extension at 72 °C for 30 s. The final extension was done at 72 °C for 2 min, and then the PCR products from the second round were stored at 4 °C. The PCR products from the second round were purified using DNA Clean Beads (Enlighten Biotech, Shanghai, China) and then quantified using the Qubit dsDNA HS Assay Kit (Thermo Fisher Scientific, Waltham, USA).

### Sequencing

The libraries originating from different samples were pooled into a mixed library with the same amount of inputs. To denature the mixed library and ensure the formation of single strands, the denaturation temperature was set as 95 °C for 3 min. The single-stranded cyclization reaction solution, digestion reaction solution, and termination solution were added sequentially using the MGIEasy Circularization Kit (MGI Tech Co., Ltd, Shenzhen, China). The cyclized products were purified, and the purified products were quantified according to the instructions. Then, the Sequencing Reagent Kit (MGI Tech Co., Ltd, Shenzhen, China) was utilized to prepare DNA nanoballs (DNB) and DNB loading system according to the manufacturer’s guidelines. The DNB concentration of 8–40 ng/µL was considered acceptable. Sequencing was conducted on the DNBSEQ-G99RS platform (MGI Tech Co., Ltd., Shenzhen, China) using the Single-End 400 bp (SE400) strategy.

### Forensic statistical analysis

In the present study, the calling of STRs was performed using STRait Razor 3.0 [38]. The quality of the obtained sequencing data of 52 STRs in the Baoan group was assessed by calculating metrics such as depth of coverage (DoC). The forensic efficacy of 52 STRs was assessed using Cervus v3.0.7 software [39] and STRAF v2.1.5 software [40] for sequence polymorphisms and length polymorphisms, respectively. The allele frequency (AF) and forensic parameters, including power of discrimination (PD), match probability (MP), polymorphism information content (PIC), probability of exclusion (PE), typical paternity index (TPI), expected heterozygosity (He), and observed heterozygosity (Ho), were counted for 20 CODIS STRs and 32 non-CODIS STRs loci, respectively. The values of cumulative discrimination power (CPD) and cumulative probability of exclusion (CPE) were calculated based on the corresponding formulae. Genepop v4.7.5 software [41] was used to test the Hardy-Weinberg equilibrium (HWE) of 52 STRs in the Baoan group, and the analysis of the Linkage Disequilibrium (LD) between pairs of autosomal STR loci was carried out by STRAF v2.1.5 software [40].

Furthermore, the system performance of the amplification panel was evaluated for the kinship identification of different degrees, covering kinship pairs including parent-child (PC) pairs, full-sibling (FS) pairs, grandparent-grandchild (GC) pairs, half-sibling (HS) pairs, uncle-nephew (UN) pairs, and first cousin (FC) pairs. In this study, 1,000 pairs of genotyping data of different kinship pairs were simulated along with 1,000 pairs of unrelated individual pairs based on the allele frequencies of 52 STRs in the Baoan group, and the calculations of LR values of different kinship pairs and unrelated individual pairs were carried out by using Familias 3 software [42]. As mentioned in previous studies, the system efficacy for the identification of different kinship under different combinations of STR loci in the Baoan group was evaluated by setting different thresholds of t1 (t2) and further calculating a series of parameters, such as system efficacy, specificity, sensitivity, inconclusive value, error rate, false positive rate (FPR) and false negative rate (FNR) [43–45]. The present study also utilized the IBS method to further assess the system effectiveness of different combinations of STRs for the identification of different kinship pairs and unrelated individual pairs. On the basis of the genotyping data of the simulated pedigrees, the combined identical by state (CIBS) values for different kinship pairs and unrelated individual pairs were calculated. The histograms of the allelic frequency distributions and probability density plots of Log_10_(LR) along with the probability density distribution plots of CIBS were drawn by *R* v4.1.2 software.

## Results

### Evaluation of NGS data quality in the Baoan group

**Fig. 1:**
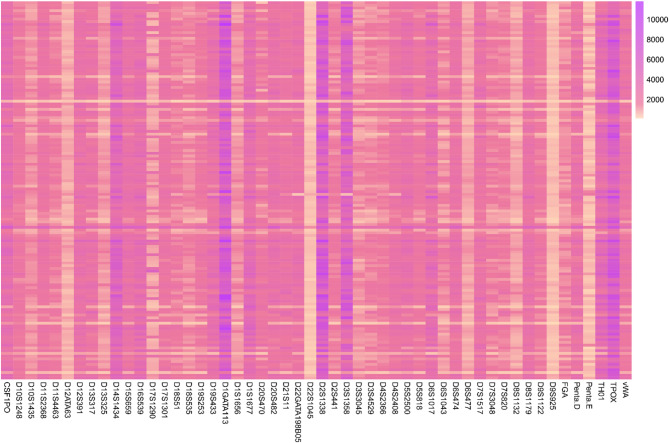
Heatmap of the sequencing depth of 52 autosomal STR loci in the 138 Baoan individuals

**Fig. 2:**
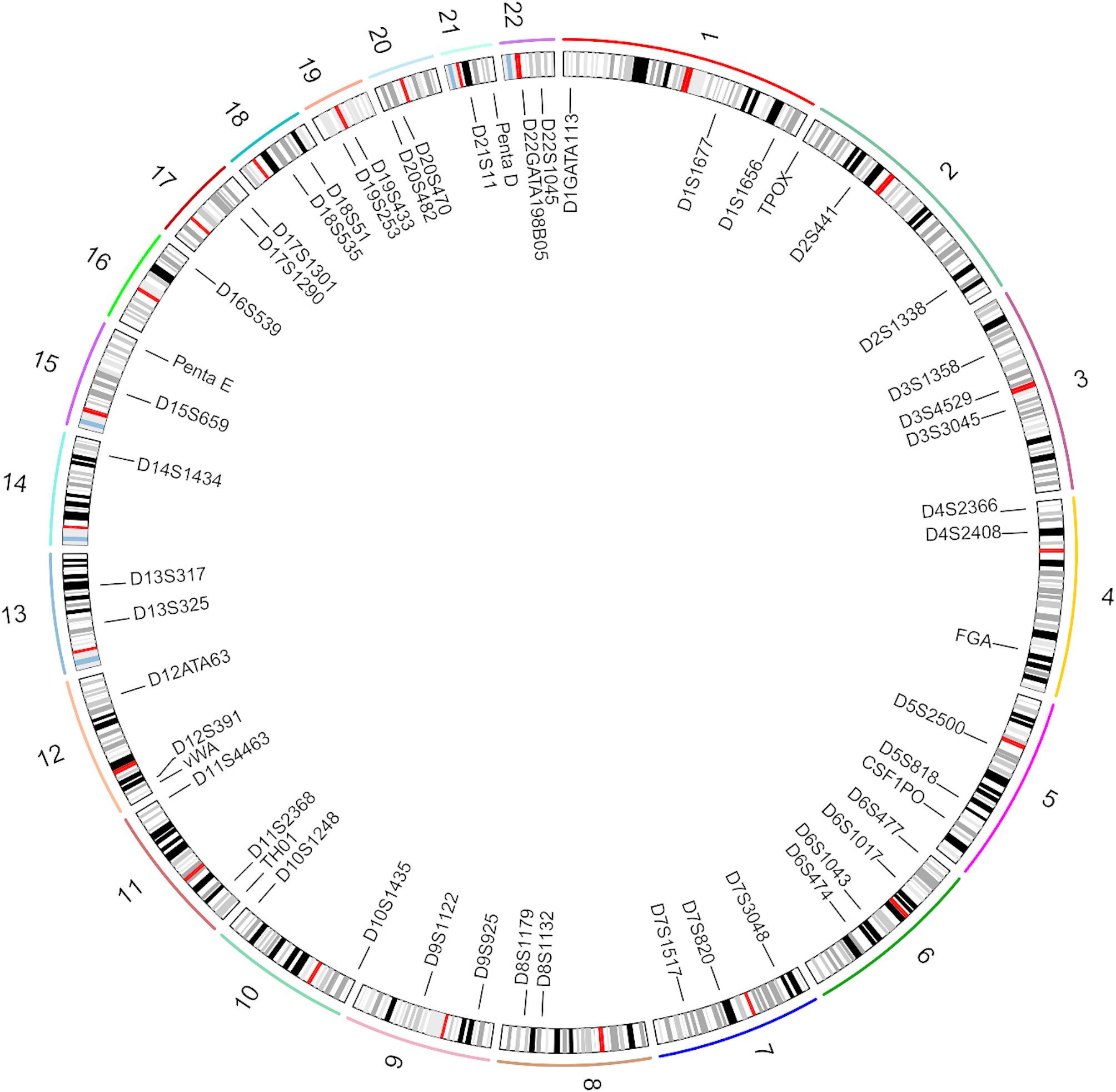
Location distributions of 52 autosomal STR loci on 22 chromosomes

This study assessed the quality of sequencing data obtained from the 52 NGS-STRs in the Baoan group, and the corresponding sequencing depth heatmap is displayed in Fig. 1. The total number of reads of the 52 STRs in 138 individuals of the Baoan group was 15,506,476, with an average read of 112,366 and a maximum read of 209,383. Among the 52 STRs in 138 individuals, the distributions of DoC values ranged from 94× to 11,830×, with the minimum value at the D9S925 locus and the maximum value at the D1GATA113 locus. Figure 2 illustrates the specific distribution locations of the 52 autosomal STRs on the chromosomes.

### HWE tests and LD analyses of 52 STRs in the Baoan group

The outcomes of pairwise LD analyses and HWE tests at 52 STRs in the Baoan group are listed in Supplementary Tables 1 and 2. In the Baoan group, the 52 STRs were conformed to HWE after Bonferroni correction (*P* > 0.05/52 = 0.00096). Besides, the 52 STRs were independent of each other and in a linkage equilibrium state following the application of multiple corrections (*P* > 0.05/1326 = 0.0000377).

### Sequence polymorphisms in the core repeat regions of 52 STRs in the Baoan group

**Fig. 3:**
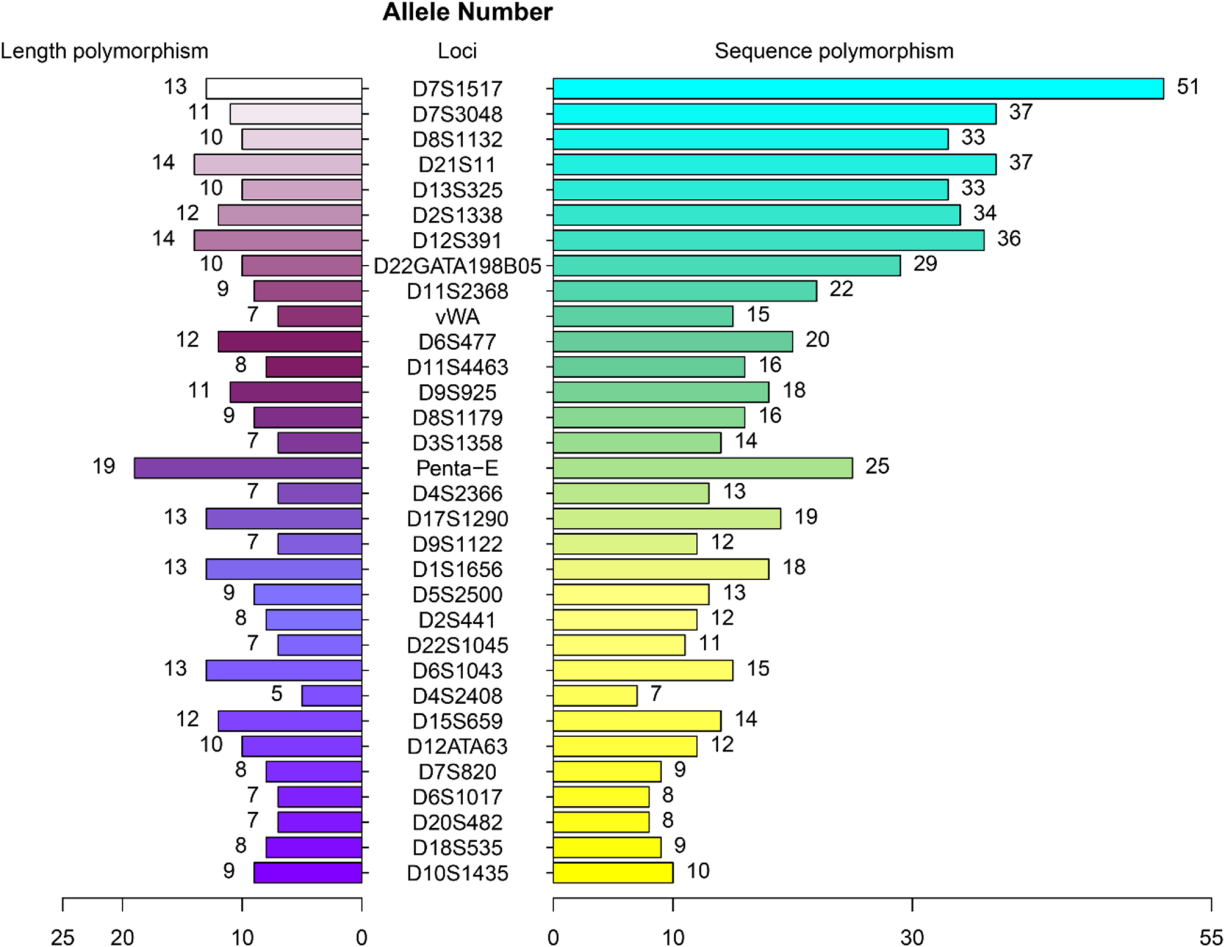
Bar graph for the numbers of STR alleles based on length polymorphisms and sequence polymorphisms

In this study, the genetic polymorphisms of 52 STRs in the Baoan group were evaluated from two dimensions of length polymorphisms and sequence polymorphisms, respectively. A total of 500 length polymorphic alleles and 805 sequence polymorphic alleles were detected in 52 STRs across 138 unrelated individuals within the Baoan group. Among them, the number of alleles discovered based on length polymorphisms and sequence polymorphisms in 20 CODIS STRs, and 32 non-CODIS STRs were 192, 295; and 308, 510, respectively. Among the 52 STRs, 32 showed an increase in the number of alleles based on their sequence polymorphisms compared with length polymorphisms. Figure 3 exhibits these loci along with the corresponding number of alleles for each locus. Among the 20 CODIS STRs, the top five with the largest difference in the numbers of sequence polymorphic alleles versus length polymorphic alleles were D21S11, D12S391, D2S1338, vWA, and D3S1358, with the numbers of allelic differences being 23, 22, 22, 8, and 7, respectively. In the 32 non-CODIS STRs, the top five with the highest number of sequence polymorphic alleles differing from length polymorphic alleles were D7S1517, D7S3048, D8S1132, D13S325, and D22GATA198B05 loci with allele number differences of 38, 26, 23, 23, and 19, respectively.

### Allele frequencies and forensic parameters of 52 STRs in the Baoan group

The study further calculated the allele frequencies and forensic parameters of 52 STRs in the Baoan group from the perspectives of length polymorphisms and sequence polymorphisms, respectively, to further investigate the genetic polymorphisms for these autosomal STRs. The allele frequencies of 52 STRs based on length polymorphisms in the Baoan group are shown in Supplementary Table 3. In the Baoan group, the values of CPD and CPE were 1–1.7341 × 10^−58^ and 1–8.8816 × 10^−22^ in 52 STRs based on length polymorphisms, whereas they were 1–2.4399 × 10^−64^ and 1–5.7014 × 10^−25^ in 52 STRs based on sequence polymorphisms, respectively.

**Fig. 4:**
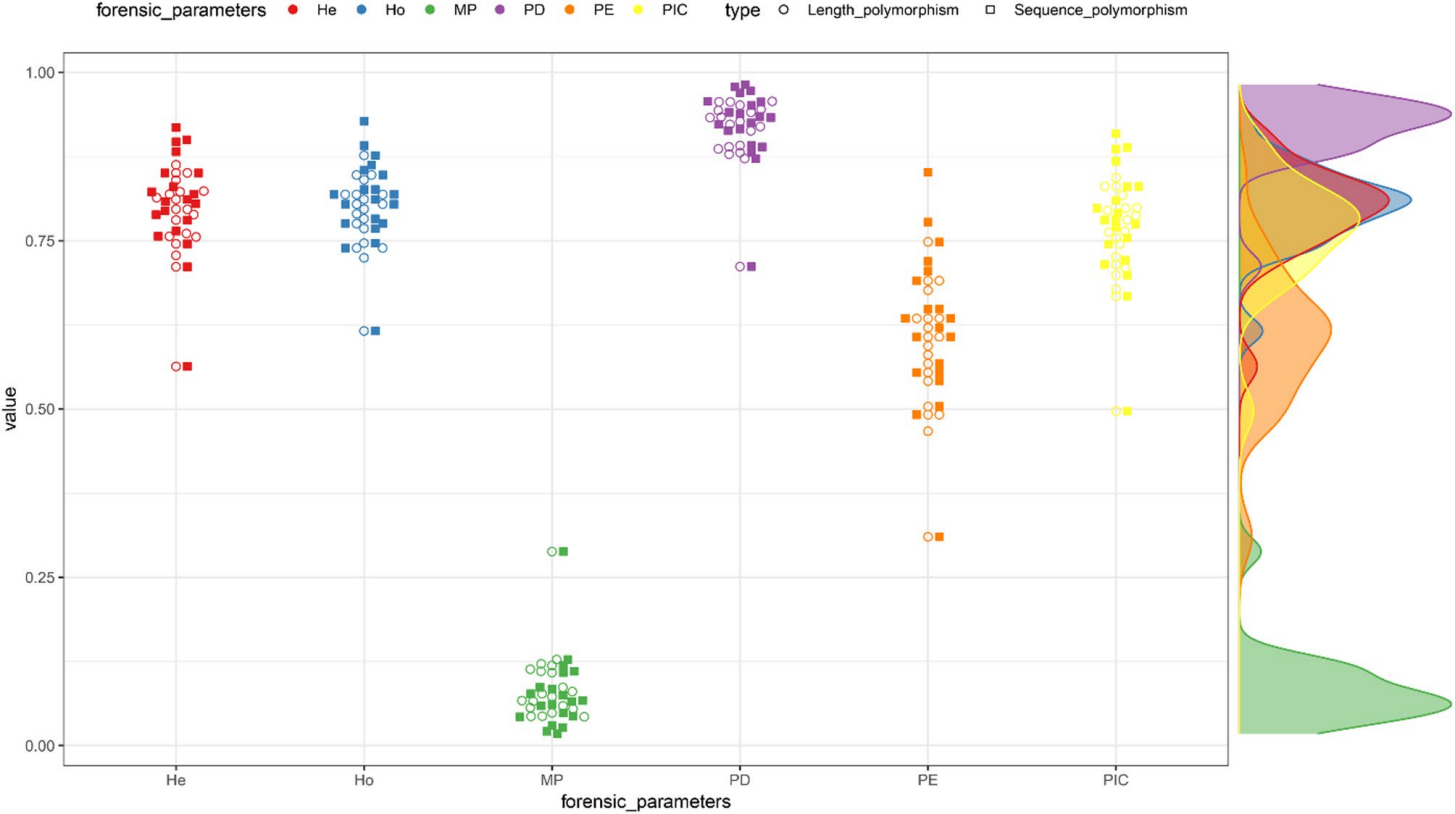
Swarm plot of forensic parameters of 20 CODIS STRs in the Baoan group based on length polymorphisms and sequence polymorphisms, respectively

**Fig. 5:**
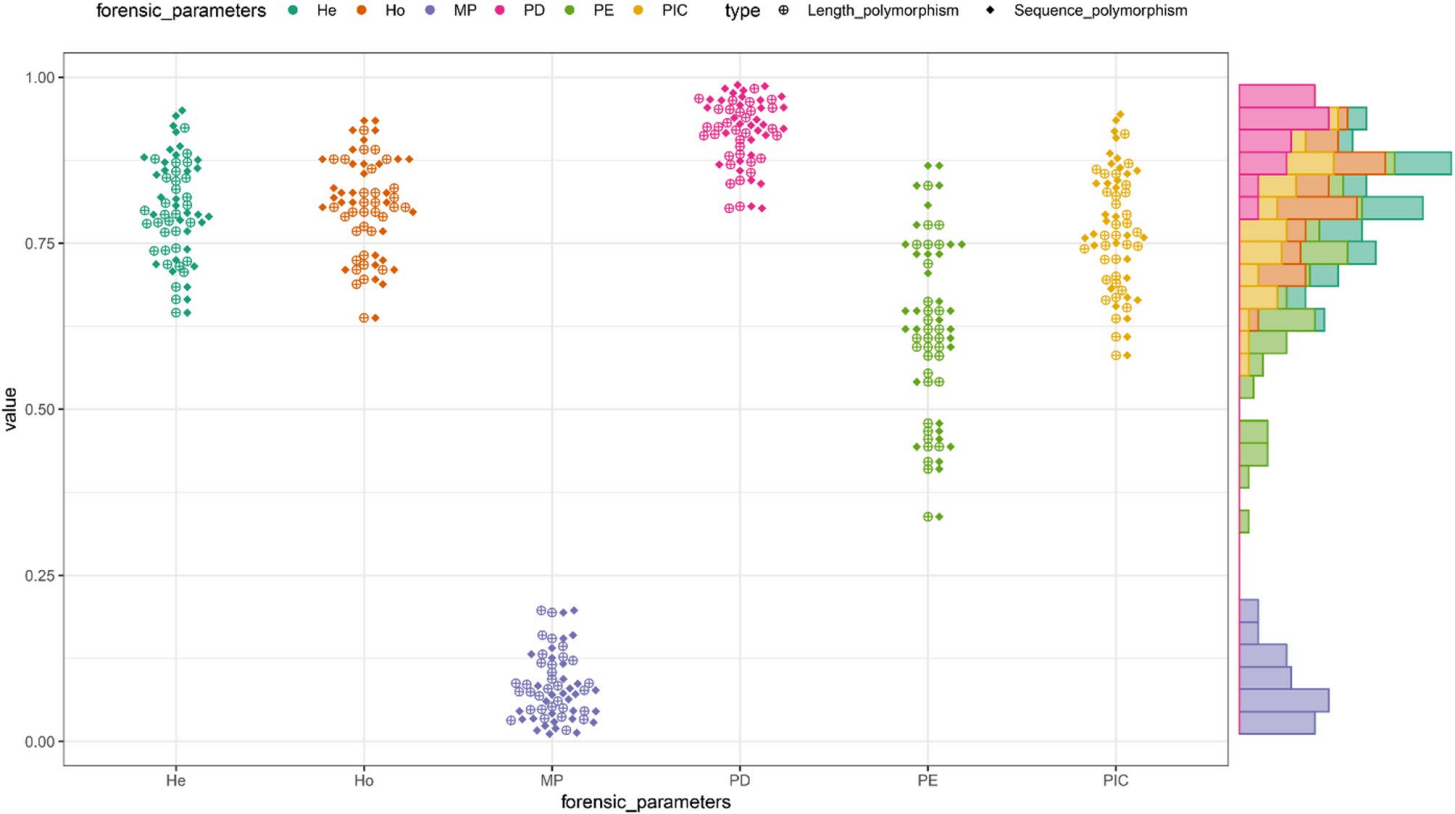
Swarm plot of forensic parameters of 32 non-CODIS STRs in the Baoan group based on length polymorphisms and sequence polymorphisms, respectively

The forensic parameters of 20 CODIS STRs and 32 non-CODIS STRs were evaluated from the perspective of length polymorphisms as well as sequence polymorphisms, respectively, and the results were displayed in Fig. 4 (20 CODIS STRs), Fig. 5 (32 non-CODIS STRs) and Supplementary Table 2. Among the 20 CODIS STRs based on length polymorphisms, the D2S1338 locus exhibited the highest values of PIC, PE, TPI, He, and Ho, while the TPOX locus recorded the lowest values of PIC, PD, PE, TPI, He, and Ho. The CPD values obtained from 20 CODIS STRs calculated based on length polymorphisms and sequence polymorphisms in the Baoan group were 1–9.0707 × 10^−23^ and 1–9.9235 × 10^−25^, respectively, while the CPE values were 1–1.5872 × 10^−08^ and 1–1.4115 × 10^−09^. Among the 32 non-CODIS STRs based on allelic sequence polymorphisms, the PIC, PD and He values of the D1GATA113 locus were the smallest, the PE, TPI and Ho values of the D6S1017 locus were the smallest, whereas the D7S1517 locus contained the largest PIC, PD, PE, TPI, He, and Ho values. For the 32 non-CODIS STRs based on length polymorphisms and sequence polymorphisms in the Baoan group, the CPD values were 1–1.9117 × 10^−36^ and 1–2.4587 × 10^−40^, respectively, with CPE values of 1–5.5956 × 10^− 14^ and 1–4.0392 × 10^−16^.

### LR values of different kinship pairs based on 52 STRs in the Baoan group

**Fig. 6:**
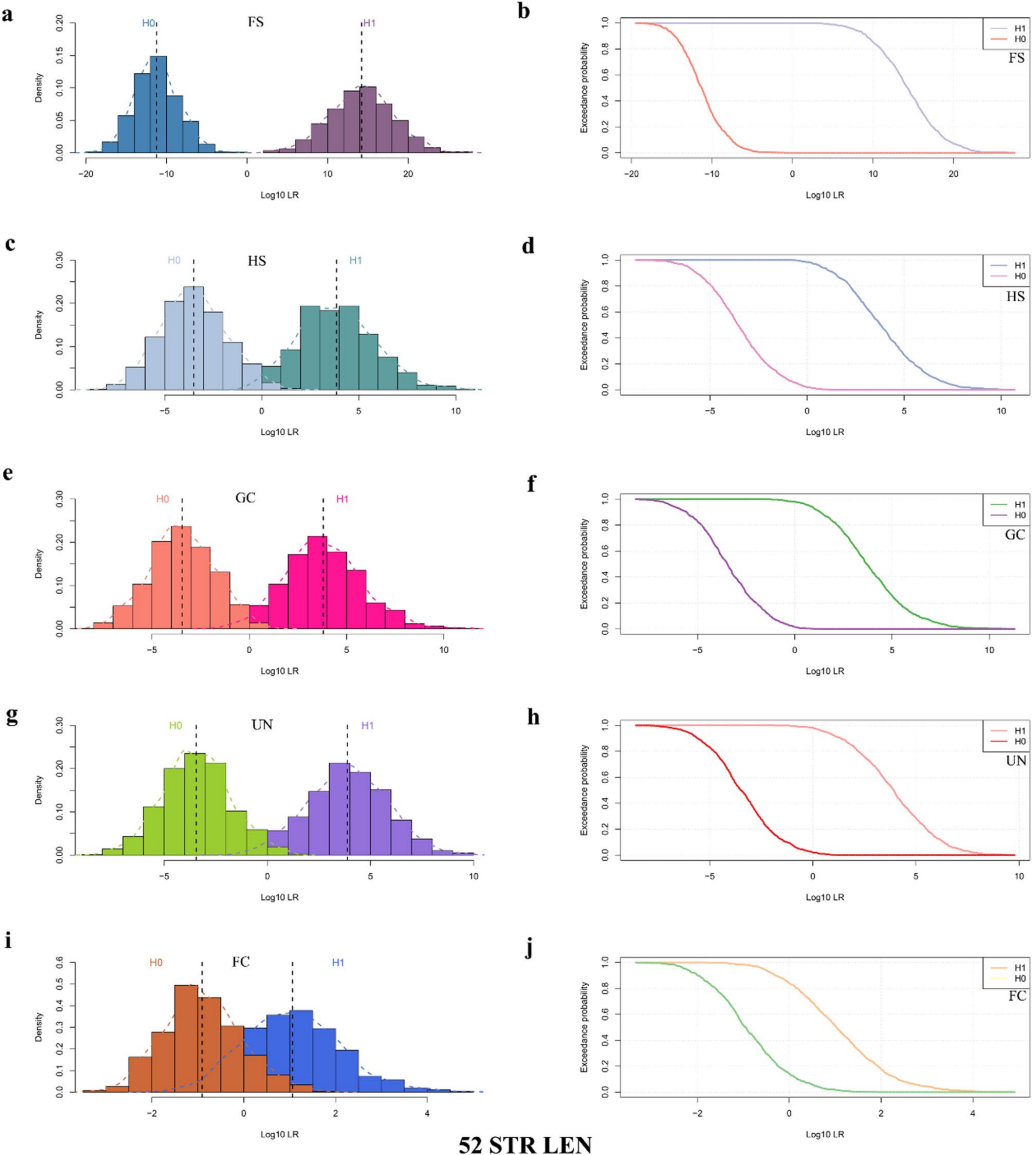
Frequency distribution histograms and expected probability density distribution curves of Log_10_(LR) for kinship pairs and unrelated individual pairs based on length polymorphisms at 52 STRs (52 STR LEN) in the Baoan group. Frequency distribution histograms of Log_10_(LR) based on length polymorphisms at 52 STRs for FS pairs and unrelated individual pairs (**a**), for HS pairs and unrelated individual pairs (**c**), for GC pairs and unrelated individual pairs (**e**), for UN pairs and unrelated individual pairs (**g**), and for FC pairs and unrelated individual pairs (**i**). Expected probability density distribution curves of Log_10_(LR) based on length polymorphisms at 52 STRs for FS pairs and unrelated individual pairs (**b**), for HS pairs and unrelated individual pairs (**d**), for GC pairs and unrelated individual pairs (**f**), for UN pairs and unrelated individual pairs (**h**), and for FC pairs and unrelated individual pairs (**j**). *LR* likelihood ratio, *FS* full-sibling, *HS* half-sibling, *GC* grandparent-grandchild, *UN* uncle-nephew, *FC* first cousin

**Fig. 7:**
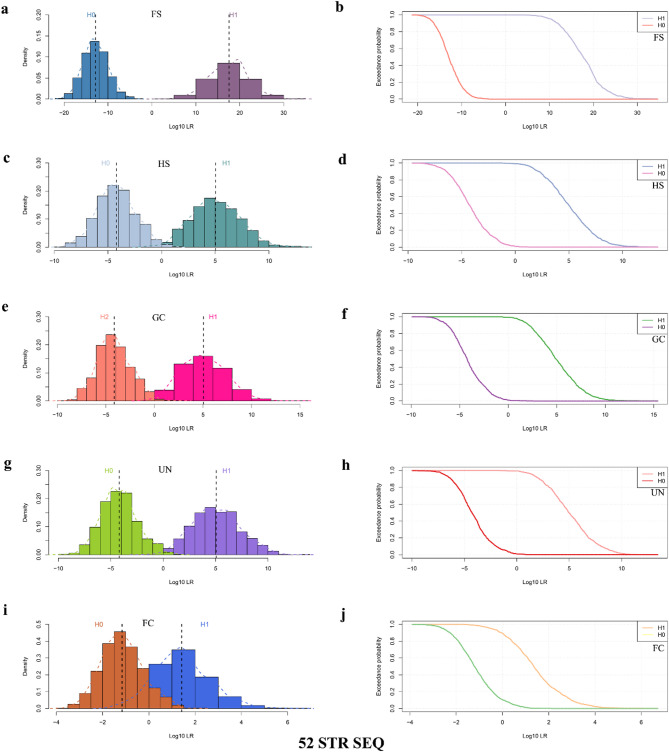
Frequency distribution histograms and expected probability density distribution curves of Log_10_(LR) for kinship pairs and unrelated individual pairs based on sequence polymorphisms at 52 STRs (52 STR SEQ) in the Baoan group. Frequency distribution histograms of Log_10_(LR) based on sequence polymorphisms at 52 STRs for FS pairs and unrelated individual pairs (**a**), for HS pairs and unrelated individual pairs (**c**), for GC pairs and unrelated individual pairs (**e**), for UN pairs and unrelated individual pairs (**g**), and for FC pairs and unrelated individual pairs (**i**). Expected probability density distribution curves of Log_10_(LR) based on sequence polymorphisms at 52 STRs for FS pairs and unrelated individual pairs (**b**), for HS pairs and unrelated individual pairs (**d**), for GC pairs and unrelated individual pairs (**f**), for UN pairs and unrelated individual pairs (**h**), and for FC pairs and unrelated individual pairs (**j**). *LR* likelihood ratio, *FS* full-sibling, *HS* half-sibling, *GC* grandparent-grandchild, *UN* uncle-nephew, *FC* first cousin

Taking the genotype data of various types of kinship pairs obtained from the simulated family pedigrees as a basis, this study further evaluated the performances of 20 CODIS STRs, 32 non-CODIS STRs and 52 STRs in identifying different levels of kinship under the dimensions of length polymorphisms and sequence polymorphisms, respectively. Supplementary Table 4 exhibits the LR values of different kinship pairs and unrelated individual pairs for the simulated tests in various combinations of STR loci. Supplementary Figs. S1–S4 and Figs. 6 and 7 respectively display the frequency distribution histograms of Log_10_(LR) and expected probability density distribution curves for different kinship pairs and unrelated individual pairs based on the length polymorphisms and sequence polymorphisms of the 20 CODIS STRs, 32 non-CODIS STRs, and 52 STRs, respectively. It can be observed that, both in the length polymorphisms and sequence polymorphisms, the LR values based on 52 STRs had no overlap between the FS pairs and the corresponding unrelated individual pairs, and there was a small range of overlap between different second-degree kinship (GC, HS, UN) pairs and the corresponding unrelated individual pairs. Moreover, with an increasing kinship degree, the range of overlap of LR values between kinship pairs and unrelated individual pairs further increased, and the LR values overlapped to a larger extent between the third-degree kinship (FC) pairs and the corresponding unrelated individual pairs.

### Assessment of the system efficacy of different kinship identifications based on 52 STRs in the Baoan group

Using the LR method, the system effectiveness of the 20 CODIS STRs, 32 non-CODIS STRs and 52 STRs based on length polymorphisms and sequence polymorphisms for the identifications of different kinship were evaluated by setting different thresholds, and the outcomes of sensitivity, specificity, FPR, FNR, inconclusive value, error rate, and effectiveness were exhibited in Supplementary Tables 5 and 6 and Tables 1 and 2, respectively.

**Table 1:** System power of 52 STRs based on length polymorphisms for simulated tests of different kinships in the Baoan group

Relationship	t1	t2	Sensitivity	Specificity	FPR	FNR	Inconclusiveness	Error rate	Effectiveness
First degree									
PC	1	− 1	1.0000	1.0000	0.0000	0.0000	0.0000	0.0000	1.0000
	2	− 2	1.0000	1.0000	0.0000	0.0000	0.0000	0.0000	1.0000
	3	− 3	1.0000	1.0000	0.0000	0.0000	0.0000	0.0000	1.0000
	4	− 4	1.0000	1.0000	0.0000	0.0000	0.0000	0.0000	1.0000
FS	1	− 1	1.0000	1.0000	0.0000	0.0000	0.0000	0.0000	1.0000
	2	− 2	1.0000	0.9980	0.0000	0.0000	0.0010	0.0000	0.9990
	3	− 3	0.9980	0.9960	0.0000	0.0000	0.0030	0.0000	0.9970
	4	− 4	0.9930	0.9940	0.0000	0.0000	0.0065	0.0000	0.9935
Second degree									
HS	1	− 1	0.9310	0.9200	0.0030	0.0000	0.0730	0.0015	0.9255
	2	− 2	0.8380	0.8100	0.0000	0.0000	0.1760	0.0000	0.8240
	3	− 3	0.6440	0.6300	0.0000	0.0000	0.3630	0.0000	0.6370
	4	− 4	0.4610	0.3920	0.0000	0.0000	0.5735	0.0000	0.4265
GC	1	− 1	0.9260	0.9290	0.0010	0.0030	0.0705	0.0020	0.9275
	2	− 2	0.8230	0.7980	0.0000	0.0000	0.1895	0.0000	0.8105
	3	− 3	0.6510	0.6100	0.0000	0.0000	0.3695	0.0000	0.6305
	4	− 4	0.4370	0.3730	0.0000	0.0000	0.5950	0.0000	0.4050
UN	1	− 1	0.9240	0.9200	0.0020	0.0040	0.0750	0.0030	0.9220
	2	− 2	0.8360	0.8180	0.0000	0.0000	0.1730	0.0000	0.8270
	3	− 3	0.6890	0.6050	0.0000	0.0000	0.3530	0.0000	0.6470
	4	− 4	0.4760	0.3700	0.0000	0.0000	0.5770	0.0000	0.4230
Third degree									
FC	1	− 1	0.5150	0.4840	0.0190	0.0170	0.4825	0.0180	0.4995
	2	− 2	0.1790	0.0980	0.0000	0.0000	0.8615	0.0000	0.1385
	3	− 3	0.0450	0.0040	0.0000	0.0000	0.9755	0.0000	0.0245
	4	− 4	0.0080	0.0000	0.0000	0.0000	0.9960	0.0000	0.0040

*PC* parent-child, *FS* full-sibling, *HS* half-sibling, *GC* grandparent-grandchild, *UN* uncle-nephew, *FC* first cousin, *FPR* false positive rate, *FNR* false negative rate, t refers to judgment threshold and is denoted as t = Log_10_(LR)

**Table 2:** System power of 52 STRs based on sequence polymorphisms for simulated tests of different kinships in the Baoan group

Relationship	t1	t2	Sensitivity	Specificity	FPR	FNR	Inconclusiveness	Error rate	Effectiveness
First degree									
PC	1	− 1	1.0000	1.0000	0.0000	0.0000	0.0000	0.0000	1.0000
	2	− 2	1.0000	1.0000	0.0000	0.0000	0.0000	0.0000	1.0000
	3	− 3	1.0000	1.0000	0.0000	0.0000	0.0000	0.0000	1.0000
	4	− 4	1.0000	1.0000	0.0000	0.0000	0.0000	0.0000	1.0000
FS	1	− 1	1.0000	1.0000	0.0000	0.0000	0.0000	0.0000	1.0000
	2	− 2	0.9990	1.0000	0.0000	0.0000	0.0005	0.0000	0.9995
	3	− 3	0.9990	1.0000	0.0000	0.0000	0.0005	0.0000	0.9995
	4	− 4	0.9990	0.9980	0.0000	0.0000	0.0015	0.0000	0.9985
Second degree									
HS	1	− 1	0.9740	0.9620	0.0020	0.0030	0.0295	0.0025	0.9680
	2	− 2	0.9100	0.8810	0.0000	0.0000	0.1045	0.0000	0.8955
	3	− 3	0.8150	0.7630	0.0000	0.0000	0.2110	0.0000	0.7890
	4	− 4	0.6700	0.5600	0.0000	0.0000	0.3850	0.0000	0.6150
GC	1	− 1	0.9720	0.9580	0.0030	0.0000	0.0335	0.0015	0.9650
	2	− 2	0.9160	0.8800	0.0010	0.0000	0.1015	0.0005	0.8980
	3	− 3	0.7960	0.7590	0.0000	0.0000	0.2225	0.0000	0.7775
	4	− 4	0.6530	0.5660	0.0000	0.0000	0.3905	0.0000	0.6095
UN	1	− 1	0.9720	0.9510	0.0010	0.0020	0.0370	0.0015	0.9615
	2	− 2	0.9150	0.8950	0.0000	0.0000	0.0950	0.0000	0.9050
	3	− 3	0.8130	0.7760	0.0000	0.0000	0.2055	0.0000	0.7945
	4	− 4	0.6640	0.5560	0.0000	0.0000	0.3900	0.0000	0.6100
Third degree									
FC	1	− 1	0.6300	0.5910	0.0120	0.0160	0.3755	0.0140	0.6105
	2	− 2	0.2810	0.1700	0.0010	0.0000	0.7740	0.0005	0.2255
	3	− 3	0.0940	0.0140	0.0000	0.0000	0.9460	0.0000	0.0540
	4	− 4	0.0230	0.0000	0.0000	0.0000	0.9885	0.0000	0.0115

*PC* parent-child, *FS* full-sibling, *HS* half-sibling, *GC* grandparent-grandchild, *UN* uncle-nephew, *FC* first cousin, *FPR* false positive rate, *FNR* false negative rate; t refers to judgment threshold and is denoted as t = Log_10_(LR)

As shown in Table 1, at all four thresholds, the system efficacy of 52 STRs based on length polymorphisms for the identification of PC pairs was 1. With an increase in the thresholds, the system efficacy of 52 STRs for the identification of FS pairs decreased gradually from 1 (t1 = 1, t2 = − 1) to 0.9990 (t1 = 2, t2 = − 2), 0.9970 (t1 = 3, t2 = − 3), and 0.9935 (t1 = 4, t2 = − 4), while the inconclusive values increased gradually from 0 (t1 = 1, t2 = − 1) to 0.0010 (t1 = 2, t2 = − 2), 0.0030 (t1 = 3, t2 = − 3), and 0.0065 (t1 = 4, t2 = − 4). When the thresholds were set between 1 (− 1) and 4 (− 4), the system efficacy of 52 STRs for the identifications of HS, GC, and UN pairs ranged from 0.9255 (t1 = 1, t2 = − 1) to 0.4265 (t1 = 4, t2 = − 4), 0.9275 (t1 = 1, t2 = − 1) to 0.4050 (t1 = 4, t2 = − 4), and 0.9220 (t1 = 1, t2 = − 1) to 0.4230 (t1 = 4, t2 = − 4), respectively. Moreover, with increasing thresholds, the sensitivity and specificity gradually decreased while the inconclusive values gradually increased. For the identification of FC pairs, the system efficacy of 52 STRs was only 0.0040 (t1 = 4, t2 = − 4), with the inconclusive value being 0.9960 (t1 = 4, t2 = − 4). As the thresholds became progressively looser, the system efficacy gradually increased to 0.0245 (t1 = 3, t2 = − 3), 0.1385 (t1 = 2 1, t2 = − 2), and 0.4995 (t1 = 1, t2 = − 1).

Table 2 demonstrates the identification results of 52 STRs based on sequence polymorphisms. Overall, the 52 STRs based on sequence polymorphisms performed better than the 52 STRs based on length polymorphisms. From threshold 1 (− 1) to threshold 4 (− 4), 52 STRs based on sequence polymorphisms could 100% distinguish PC pairs from unrelated individual pairs with an error rate of 0. As the threshold increased, the error rate of 52 STRs for the identification of FS pairs remained 0, but the inconclusive value increased from 0 (t1 = 1, t2 = − 1) to 0.0015 (t1 = 4, t2 = − 4), sensitivity decreased from 1 (t1 = 1, t2 = − 1) to 0.9990 (t1 = 4, t2 = − 4), specificity decreased from 1 (t1 = 1, t2 = − 1) to 0.9980 (t1 = 4, t2 = − 4), and system effectiveness decreased from 1 (t1 = 1, t2 = − 1) to 0.9985 (t1 = 4, t2 = − 4). At a threshold value of 1 (− 1), for the identifications of the second-degree kinship pairs (HS, GC, UN), the 52 STRs showed a sensitivity between 0.9720 and 0.9740, specificity between 0.9510 and 0.9620, uncertainty between 0.0295 and 0.0370, and system effectiveness between 0.9615 and 0.9680. When the threshold was 4 (− 4), the sensitivity, specificity and system effectiveness decreased to between 0.6530 and 0.6700, 0.5560 and 0.5660, and 0.6095 and 0.6150, respectively, while the inconclusive values increased to between 0.3850 and 0.3905. When identifying the third-degree kinship pairs (FC), the inconclusive value was obviously higher and the system effectiveness was noticeably lower compared to the first- and second-degree kinship. Under all four thresholds, the inconclusive values were 0.3755 (t1 = 1, t2 = − 1), 0.7740 (t1 = 2, t2 = − 2), 0.9460 (t1 = 3, t2 = − 3), and 0.9885 (t1 = 4, t2 = − 4), while the system effectiveness values were 0.6105 (t1 = 1, t2 = − 1), 0.2255 (t1 = 2, t2 = − 2), 0.0540 (t1 = 3, t2 = − 3), and 0.0115 (t1 = 4, t2 = − 4), respectively.

### Effectiveness evaluation of different combinations of STR loci

**Fig. 8:**
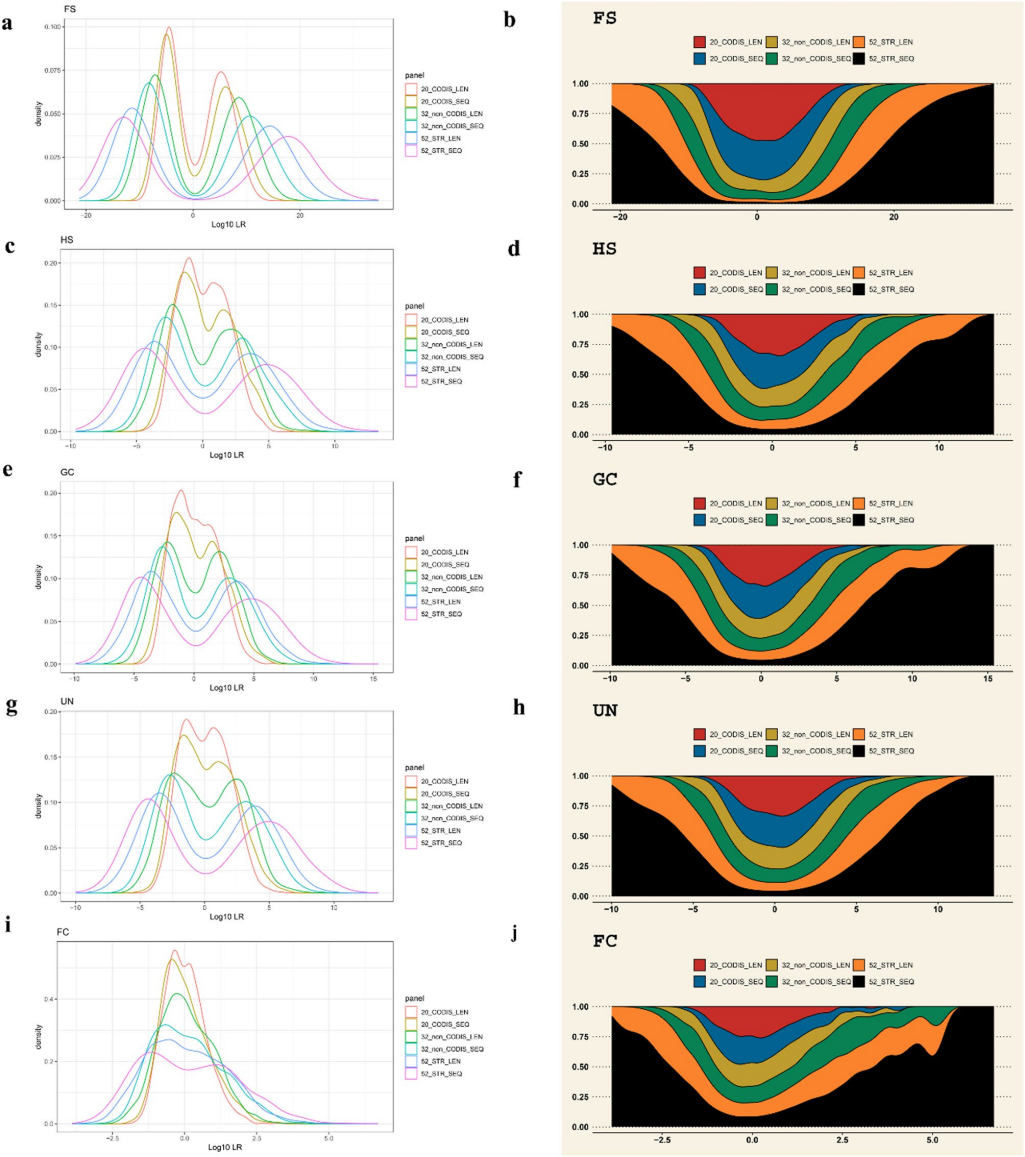
Probability density plots and area stacking diagrams of Log_10_(LR) for kinship pairs and unrelated individual pairs under different combinations of STR loci in the Baoan group. Probability density plots of Log_10_(LR) for FS pairs and unrelated individual pairs (**a**), for HS pairs and unrelated individual pairs (**c**), for GC pairs and unrelated individual pairs (**e**), for UN pairs and unrelated individual pairs (**g**), and for FC pairs and unrelated individual pairs (**i**). Area stacking diagrams of Log_10_(LR) for FS pairs and unrelated individual pairs (**b**), for HS pairs and unrelated individual pairs (**d**), for GC pairs and unrelated individual pairs (**f**), for UN pairs and unrelated individual pairs (**h**), for FC pairs and unrelated individual pairs (**j**). *LR* likelihood ratio, *FS* full-sibling, *HS* half-sibling, *GC* grandparent-grandchild, *UN* uncle-nephew, *FC* first cousin

This study further evaluated the system efficacy of different kinship identifications using the LR method with six combinations of STRs, including 20 CODIS STRs, 32 non-CODIS STRs, and 52 STRs based on length polymorphisms and sequence polymorphisms, respectively. The results of different kinship including FS, HS, GC, UN, FC pairs, and corresponding unrelated individual pairs are displayed sequentially from top to bottom in Fig. 8. Besides, the left side displayed the probability density plots of Log_10_(LR) based on different combinations of STRs with length polymorphisms and sequence polymorphisms, and the right side displayed the corresponding area stacking diagrams of Log_10_(LR). Notably, among the six combinations, the combination results of 52 STRs based on sequence polymorphisms showed the smallest overlap in the distribution of Log_10_(LR) between different kinship pairs and unrelated individual pairs. The system performance of the combination of 52 STRs based on sequence polymorphisms was higher than those of the remaining five combinations of STR loci mentioned above in the identifications of different kinship pairs. The HS, GC and UN pairs exhibited similar curve profiles and overlapping distributions, suggesting that different combinations of STR loci have limited ability to distinguish the relatives belonging to different types of the same kinship degree. Moreover, the FC pairs showed larger overlapping regions with the unrelated individual pairs, representing the lower capacity of different combinations of STR loci for the identification of third-degree kinship.

### CIBS of different combinations of STRs in different kinship pairs

**Fig. 9:**
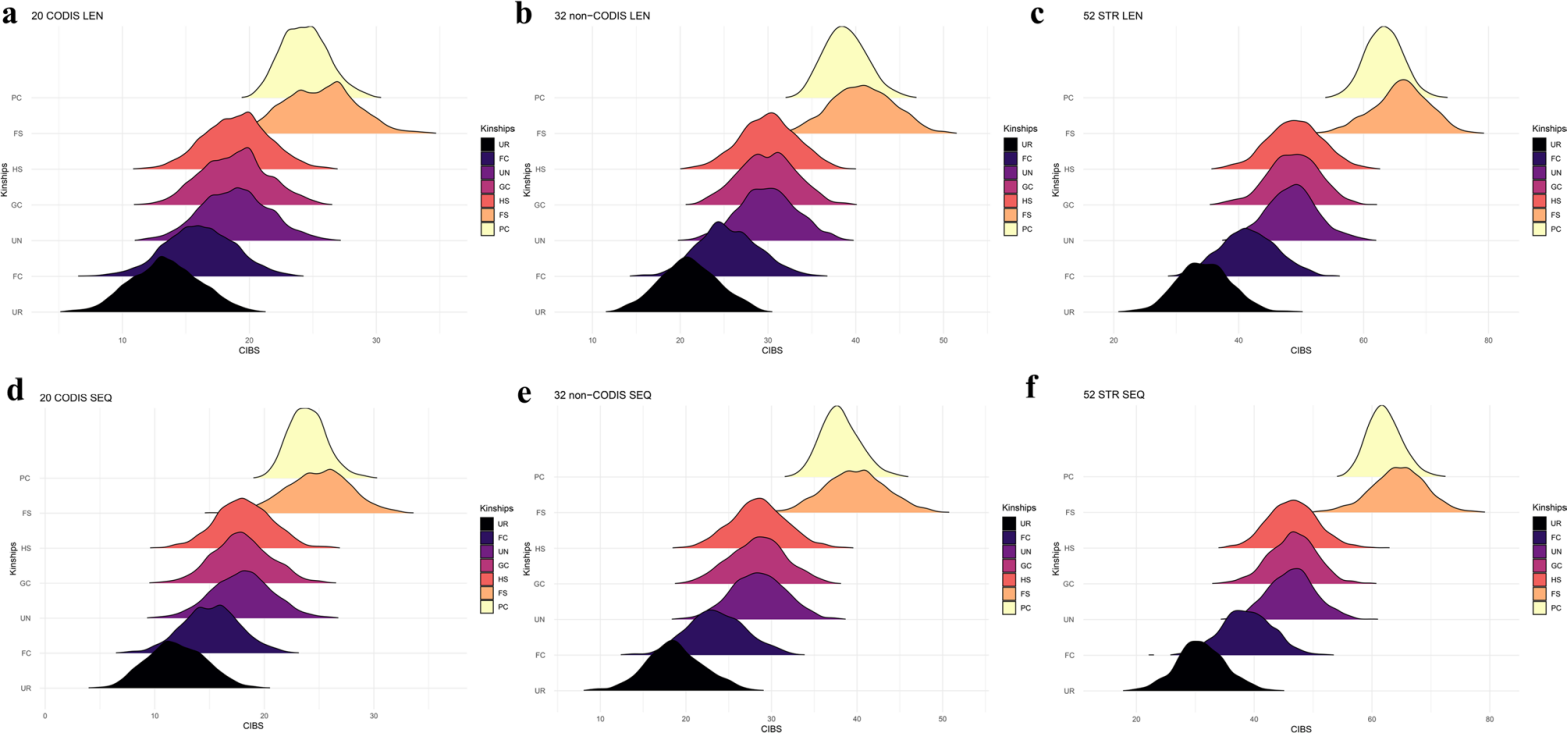
Distributions of density curves for CIBS in kinship pairs (FS, HS, GC, UN, FC) and unrelated individual pairs under different combinations of STR loci in the Baoan group. Distributions of density curves for CIBS in kinship pairs and unrelated individual pairs based on length polymorphisms at 20 CODIS STRs (**a**), based on length polymorphisms at 32 non-CODIS STRs (**b**), based on length polymorphisms at 52 STRs (**c**), based on sequence polymorphisms at 20 CODIS STRs (**d**), based on sequence polymorphisms at 32 non-CODIS STRs (**e**), and based on sequence polymorphisms at 52 STRs (**f**). *CIBS* combined identical by state, *FS* full-sibling, *HS* half-sibling, *GC* grandparent-grandchild, *UN* uncle-nephew, *FC* first cousin

Furthermore, the distributions of CIBS in different kinship pairs and unrelated individual pairs were calculated on the basis of the genotype results of the simulated pedigrees, and the outcomes acquired from different combinations of STRs were presented in the probability density curves depicted in Fig. 9 and Supplementary Table 7. The results revealed that the highest mean value of CIBS was found in the first-degree kinship pairs, and the lowest mean value of CIBS was found in the unrelated individual pairs. The mean values of CIBS for PC, FS, HS, GC, UN, FC pairs and unrelated individual pairs of 52 STRs based on length polymorphisms and sequence polymorphisms were 63.27, 65.96, 49.04, 48.79, 48.61, 41.54, 34.48; and 62.13, 64.56, 46.59, 46.57, 46.45, 38.79, 30.98, respectively. These results indicate that the highest average CIBS values were found in the FS pairs and the lowest in the unrelated individual pairs. The distributions of CIBS for PC pairs with unrelated individual pairs and that for FS pairs with unrelated individual pairs were completely separated. The mean values of CIBS for the HS, GC, and UN pairs were larger than those of the FC pairs, and the degree of overlap among the HS, GC, and UN pairs with the unrelated individual pairs was smaller than that between the FC pairs and the unrelated individual pairs.

## Discussion

From the perspective of population genetics, the Baoan people represent a valuable subject for research. This is because its relatively small population size, historical practice of endogamy (the custom of marrying within the group), and unique demographic history may have shaped a distinctive genetic structure [35]. To characterize its genetic makeup, including the allele frequencies of forensic molecular markers such as STRs, it is crucial to establish a region-specific DNA database. Such database can have a core value for precise identification and kinship testing within a population, as genetic marker frequencies exhibit significant variations across different ethnic groups [46, 47]. In the targeted study population, the allele frequencies of particular loci formed the basis of evidence interpretation for forensic individual identification and kinship identification. Previous genetic research on the Baoan group has been limited in scope, focusing solely on genotyping a few STR loci using CE technology, and with no NGS-based data available [36]. Therefore, it is an urgent task to conduct comprehensive research using NGS technology to reveal the length and sequence polymorphisms of the Baoan group, providing more accurate and valuable population genetic datasets for the forensic application on complex kinship relationships.

STR sequence polymorphisms refer to variations in base composition within the core repeat units and flanking regions of STRs. While CE technology only detects alleles based on fragment length differences, NGS technology enables full sequencing analysis of each STR allele, revealing hidden sequence variations such as single nucleotide polymorphism (SNP), insertion, deletion, microvariant, et al. [48–51]. For example, at the D2S1338 locus, CE technology can only detect the length allele “17 repeats”, whereas NGS technology can further distinguish length allele with two different sequence variations: “[GGAA]_10_ [GGCA]_7_” and “[GGAA]_11_ [GGCA]_6_”. In the present study, 52 STRs in 138 individuals of the Baoan group were detected using NGS technology. Compared with length polymorphisms, the number of alleles obtained based on sequence polymorphisms was increased by 305, comprising an increase rate of 61.00%. Among the 20 CODIS STR loci, the number of alleles was increased at 8 STR loci, and the total number of alleles was increased from 192 to 295, equivalent to an increase rate of 53.65%. The three STR loci with the most marked increase rates were D2S1338 (183.33%), D21S11 (164.29%), and D12S391 (157.14%). As for the 32 non-CODIS STR loci, 22 loci showed increase in the number of detected alleles, and the total number of alleles was increased from 308 to 510, an increase rate of 65.58%. It is worth noting that the increase in some alleles was over 200%, such as the D7S1517 and D7S3048 loci, with growth rates of 292.31% and 236.36%, respectively. The frequency distributions of repeat structures at the same STR locus vary across different populations. In the present study, among the 138 Baoan individuals, the predominant repeat structure at the D21S11 locus is [TCTA]_4−8_[TCTG]_5−7_[TCTA]_3_TA[TCTA]_3_TCA[TCTA]_2_TCCATA[TCTA]_8−13_, while at the D7S1517 locus, it was [CTTT]_2_ [GTTT]_1_ [CTTT]_1–2_ [GTTT]_1–6_ [CTTT]_8–19_.

The PIC, MP, PD, PE, TPI, He, and Ho values are commonly used forensic parameters in the field of population genetics. Overall, in the majority of STRs, the PIC, PD, PE, TPI, He, and Ho values of STR sequence polymorphisms increased to different degrees compared to length polymorphisms, whereas the MP values exhibited different degrees of decrease. The variations in the 7 forensic parameters also reflect the increase in forensic detection efficacy brought about by the information of sequence polymorphisms to some extent. In a study of the Baoan group, Sun et al. [36] detected 150 alleles at 15 STRs, and obtained a combined match probability (CPM) of 7.9243 × 10^−18^, CPD of 0.999999999999999992076, and CPE of 0.999998259. Meanwhile, in this study, a total of 1305 alleles were obtained from 52 STRs based on length polymorphisms and sequence polymorphisms. The CPD (1–1.7341 × 10^−58^) and CPE (1–8.8816 × 10^−22^) obtained from 52 STRs based on length polymorphisms and the CPD (1–2.4399 × 10^−64^) and CPE (1–5.7014 × 10^−25^) obtained from 52 STRs based on sequence polymorphisms were both larger than those in Sun et al. [36], suggesting that the present amplification system has higher forensic detection efficacy for the Baoan group.

The emergence of sequence polymorphism information has led to an increase in forensic detection efficacy compared with the genotyping methods based on length polymorphisms [25, 52–54]. In the present study, an increase in allelic polymorphism based on sequence variations was also found in the Baoan group, where the CPM value (9.0707 × 10^−23^) of 20 CODIS STRs based on length polymorphisms was greater than that (9.9235 × 10^−25^) of 20 CODIS STRs based on sequence polymorphisms. These findings further confirm the hypothesis that NGS-based sequence analysis could enhance the allele polymorphisms of STRs, and reveal hidden genetic diversity inaccessible to CE, thereby improving the efficacy of the forensic detection system. The potential performance improvement obtained by the identification of sequence information based on NGS technology has a promising prospect for future forensic application.

Moreover, this study evaluated the distributions of Log_10_(LR) and CIBS values in different kinship pairs and unrelated individual pairs, then further assessed the combination efficacy of different STR loci for the identification of kinship from the perspectives of sequence polymorphisms and length polymorphisms, respectively. It was found that the ability of the detection system to discriminate between different classes of kinship pairs and unrelated individual pairs rose by a certain degree as the number of loci increased. When length polymorphisms were taken into account and the threshold was set to 4 (− 4), the system efficacy of 52 STRs increased from 0.9920 (1) to 1 for the identification of PC pairs, it increased from 0.6530 (0.9365) to 0.9935 for FS pairs, it increased from 0.0125 (0.1110) to 0.4265 for HS pairs, it increased from 0.0140 (0.1095) to 0.4050 for GC pairs, it increased from 0.0125 (0.1020) to 0.4230 for UN pairs, and it increased from 0 (0) to 0.0040 for FC pairs, as compared to the use of 20 CODIS STRs (or 32 non-CODIS STRs) alone. It was discovered that the highest identification efficacy in different kinship pairs was achieved by the combination of 52 STRs based on sequence polymorphisms. Furthermore, the acquisition of sequence variable information increased the polymorphisms of STR genetic markers, which enhanced the performance in complex kinship analysis to a certain extent, consistent with previous studies [55, 56]. When the absolute value of the threshold was set to 1 (or 4), compared to 52 STRs based on length polymorphisms, the system efficacy of 52 STRs based on sequence polymorphisms was improved by 0 (0.0050) for the identification of FS pairs, by 0.0425 (0.1885), 0.0375 (0.2045), and 0.0395 (0.1870) for the identifications of HS, GC, and UN pairs, respectively, and by 0.1110 (0.0075) for the identification of FC pairs. The results of this study further demonstrate that, on the one hand, increasing the number of genetic markers and obtaining sequence information can significantly improve the systemic efficacy of the detection panel for the identification of complex relationships, and on the other hand, exploring other combinations of systems with higher effectiveness is important in the current forensic practice of complex kinship analysis.

Regarding the effectiveness of NGS-STRs in resolving various kinship relationships, our findings revealed a hierarchical pattern. Overall, the system effectiveness of the present amplification system was high for the identification of first-degree kinship, but relatively limited for the identifications of second-degree or higher-degree kinship. This finding underscores the immediate practical value of this amplification system for routine PC and FS identifications in forensic cases, with the test results exhibiting exceptionally high reliability. Regarding second-degree kinship, the results demonstrated that the sequence polymorphisms yielded more than 18% absolute improvement. This indicates that integrating NGS-STRs can enhance the success rate of second-degree kinship cases, providing valuable evidence for immigration, inheritance, and missing person investigations. However, the overlapping distributions of LR values among different types of second-degree kinship show that the distinguishing relationships between them still remains challenging, even with the use of NGS-STRs. For third-degree kinship, the effectiveness of this amplification system was significantly low, with a high incidence of inconclusive results. This reveals the existing limitations of the current NGS-STRs system and underscores the need to employ strategies that combine STRs with other marker types or involve additional relatives when dealing with distant kinship relationships.

The findings of this study reveal multiple benefits for forensic casework. Firstly, the study provide a comprehensive population genetic database of 52 NGS-STRs for the Baoan people, which is crucial for precise LR calculations when handling cases involving individuals from this group. Secondly, this study further validates the practical advantages of the NGS-STR detection system compared to the traditionally used CE system: the ability to obtain massive amounts of sequence information through a single test makes NGS technology especially valuable for identifying complex kinship. Finally, the quantitative data for each type of kinship obtained in this study provides forensic geneticists with useful guidance for establishing appropriate interpretation criteria.

This study has several limitations to be considered when interpreting its findings. First, though the sample size was sufficient for preliminary allele frequency estimation, future research needs to expand the sample size to capture rarer alleles and further refine the population statistical analysis. Second, it was conducted exclusively on the Baoan group, and hence the obtained results may not be generalizable to other ethnic groups due to the differences in genetic structure. Future cross-population validation is required to assess the broader applicability of this amplification system. Third, the kinship analysis in this study was based solely on simulated pedigree data derived from allele frequencies. While simulated data are acceptable, they cannot fully replicate real-world challenges such as DNA degradation, samples contamination, or rare allelic variation. To further confirm the obtained conclusions, subsequent research should validate the system using real pedigree samples from known kinship pairs and forensic samples. Finally, the system exhibited low efficiency in third-degree kinship, limiting its application in the identification of distant relationships. Introducing additional markers, such as microhaplotypes or SNPs, into future multiplex amplification systems is a crucial step in improving system efficacy.

## Conclusions

In summary, this study demonstrates that integrating sequence polymorphisms could significantly enhance the discriminatory power of STR loci in the Baoan group, overcoming the limitations of the traditional length-based genotyping method. The NGS-STR system demonstrated high system efficacy in identifying first-degree kinship. When combined with sequence variation analysis, the detection system achieved significantly enhanced performance in identifying second-degree kinship. However, the identification of third-degree kinship remains challenging and requires the further expansion of different marker numbers. These above findings underscore the critical value of NGS in forensic genetics, providing a more robust framework for complex kinship analysis. Future work should focus on developing multiplex detection systems for more loci and integrating multiple types of genetic markers to further enhance the power of complex kinship analysis in forensic practice.

## Supplementary Information

Supplementary Material 1.

Supplementary Material 2.

## Data Availability

The datasets used during the current study are available from the corresponding author on reasonable request.

## Abbreviations

LR: Likelihood ratio
IBS: Identical by state
STR: Short tandem repeat
CE: Capillary electrophoresis
CODIS: Combined DNA index system
NGS: Next-generation sequencing
MPS: Massively parallel sequencing
DNB: DNA nanoball
SE400: Single-end 400
DoC: Depth of coverage
AF: Allele frequency
PD: Power of discrimination
MP: Match probability
PIC: Polymorphism information content
PE: Probability of exclusion
TPI: Typical paternity index
He: Expected heterozygosity
Ho: Observed heterozygosity
CPD: Cumulative discrimination power
CPE: Cumulative probability of exclusion
HWE: Hardy-Weinberg equilibrium
LD: Linkage disequilibrium
PC: Parent–child
FS: Full-sibling
GC: Grandparent–grandchild
HS: Half-sibling
UN: Uncle–nephew
FC: First cousin
FPR: False positive rate
FNR: False negative rate
CIBS: Combined identical by state
CPM: Combined match probability
SNP: Single nucleotide polymorphism
