# Supplementary material for: Forensic utilization of NGS-STRs and evaluation of system efficacy for different kinship identifications
Source: Hum Genomics. 2025 Dec 24;19:151. doi: 10.1186/s40246-025-00858-y (PMC12729311; doi:10.1186/s40246-025-00858-y)
Supplement: Supplementary file 1 — Supplementary Material 1. [file 40246_2025_858_MOESM1_ESM.docx]

**Forensic application of NGS-STRs and evaluation of system efficacy for different kinship identifications**


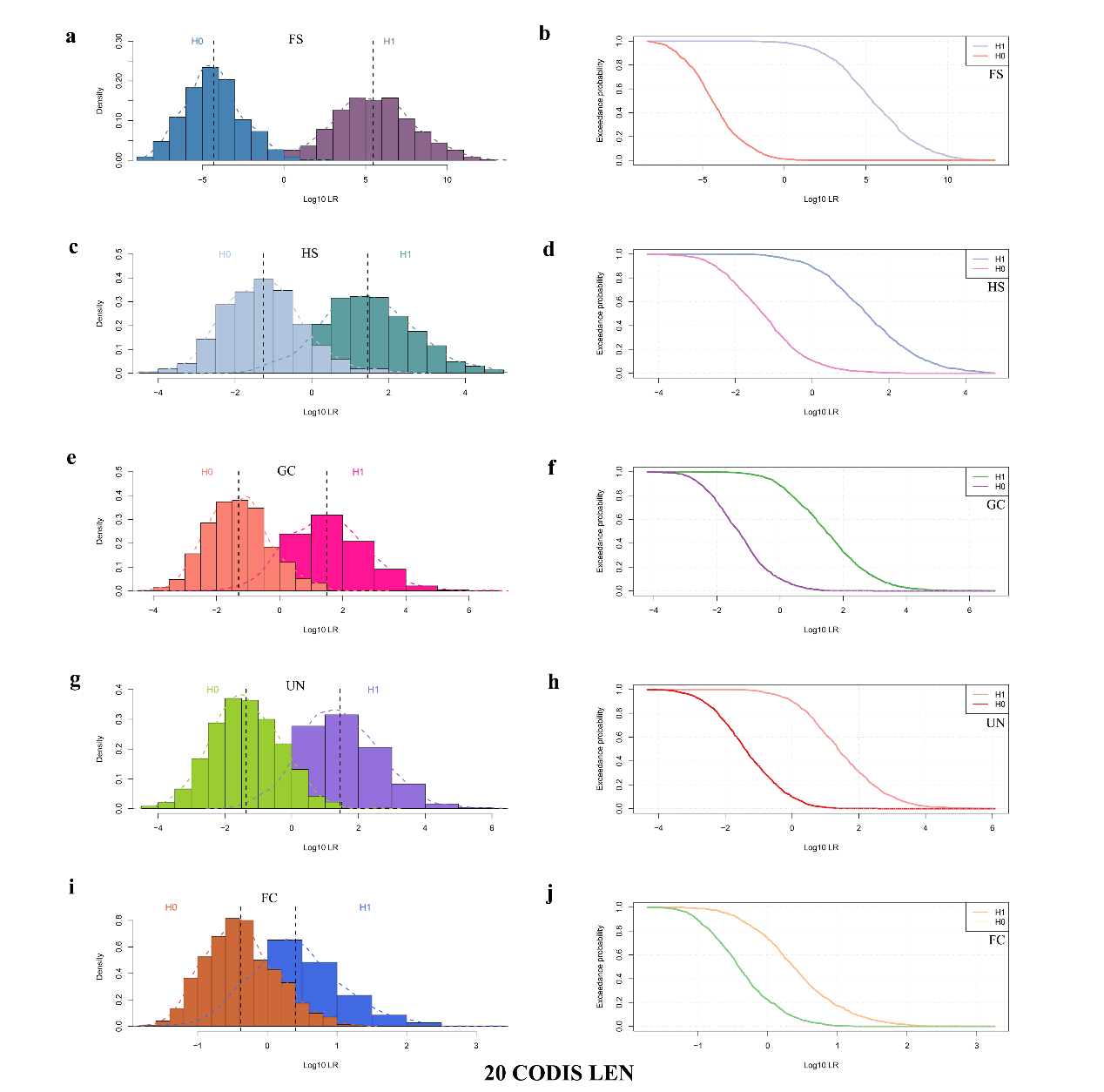


**Supplementary Fig. 1.** Frequency distribution histograms and expected probability density distribution curves of Log_10_(LR) for kinship pairs and unrelated individual pairs based on length polymorphisms at 20 CODIS STRs (20 CODIS LEN) in the Baoan group. Frequency distribution histograms of Log_10_(LR) based on length polymorphisms at 20 CODIS STRs for FS pairs and unrelated individual pairs (a), for HS pairs and unrelated individual pairs (c), for GC pairs and unrelated individual pairs (e), for UN pairs and unrelated individual pairs (g), and for FC pairs and unrelated individual pairs (i). Expected probability density distribution curves of Log_10_(LR) based on length polymorphisms at 20 CODIS STRs for FS pairs and unrelated individual pairs (b), for HS pairs and unrelated individual pairs (d), for GC pairs and unrelated individual pairs (f), for UN pairs and unrelated individual pairs (h), and for FC pairs and unrelated individual pairs (j). LR, likelihood ratio; FS, full-sibling; HS, half-sibling; GC, grandparent-grandchild; UN, uncle-nephew; FC, first cousin.


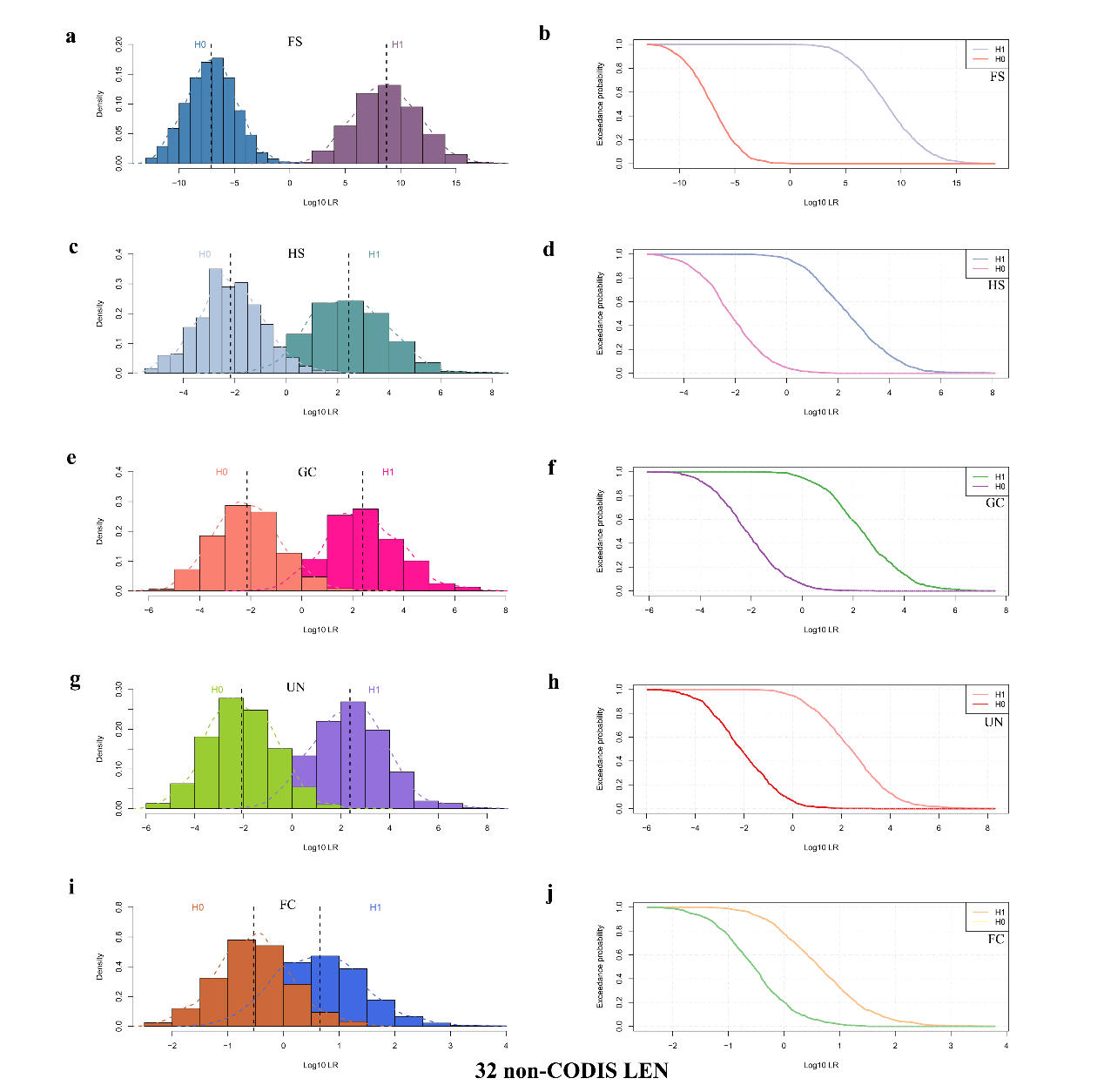


**Supplementary Fig. 2.** Frequency distribution histograms and expected probability density distribution curves of Log_10_(LR) for kinship pairs and unrelated individual pairs based on length polymorphisms at 32 non-CODIS STRs (32 non-CODIS LEN) in the Baoan group. Frequency distribution histograms of Log_10_(LR) based on length polymorphisms at 32 non-CODIS STRs for FS pairs and unrelated individual pairs (a), for HS pairs and unrelated individual pairs (c), for GC pairs and unrelated individual pairs (e), for UN pairs and unrelated individual pairs (g), and for FC pairs and unrelated individual pairs (i). Expected probability density distribution curves of Log_10_(LR) based on length polymorphisms at 32 non-CODIS STRs for FS pairs and unrelated individual pairs (b), for HS pairs and unrelated individual pairs (d), for GC pairs and unrelated individual pairs (f), for UN pairs and unrelated individual pairs (h), and for FC pairs and unrelated individual pairs (j). LR, likelihood ratio; FS, full-sibling; HS, half-sibling; GC, grandparent-grandchild; UN, uncle-nephew; FC, first cousin.


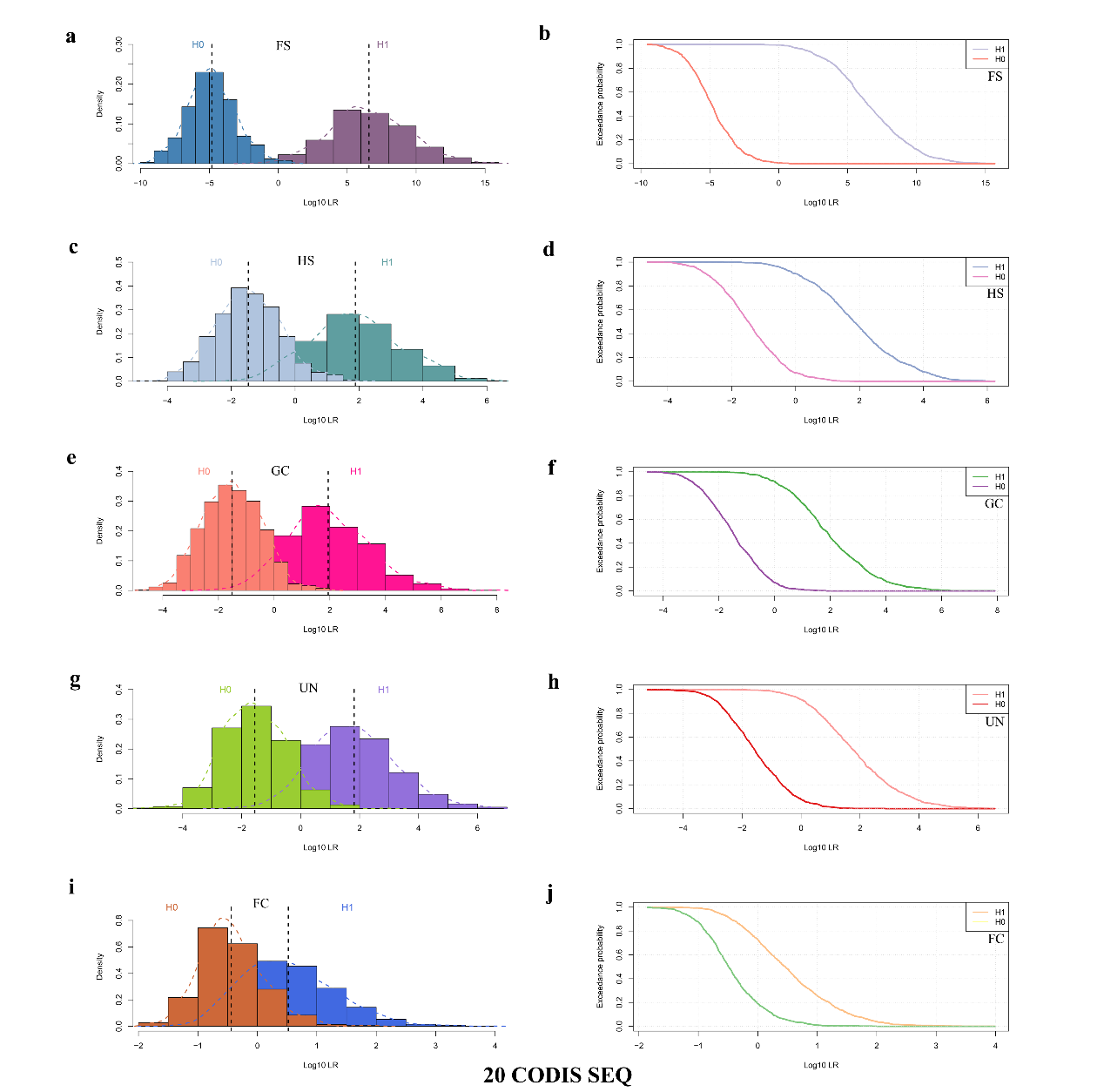


**Supplementary Fig. 3.** Frequency distribution histograms and expected probability density distribution curves of Log_10_(LR) for kinship pairs and unrelated individual pairs based on sequence polymorphisms at 20 CODIS STRs (20 CODIS SEQ) in the Baoan group. Frequency distribution histograms of Log_10_(LR) based on sequence polymorphisms at 20 CODIS STRs for FS pairs and unrelated individual pairs (a), for HS pairs and unrelated individual pairs (c), for GC pairs and unrelated individual pairs (e), for UN pairs and unrelated individual pairs (g), and for FC pairs and unrelated individual pairs (i). Expected probability density distribution curves of Log_10_(LR) based on sequence polymorphisms at 20 CODIS STRs for FS pairs and unrelated individual pairs (b), for HS pairs and unrelated individual pairs (d), for GC pairs and unrelated individual pairs (f), for UN pairs and unrelated individual pairs (h), and for FC pairs and unrelated individual pairs (j). LR, likelihood ratio; FS, full-sibling; HS, half-sibling; GC, grandparent-grandchild; UN, uncle-nephew; FC, first cousin.


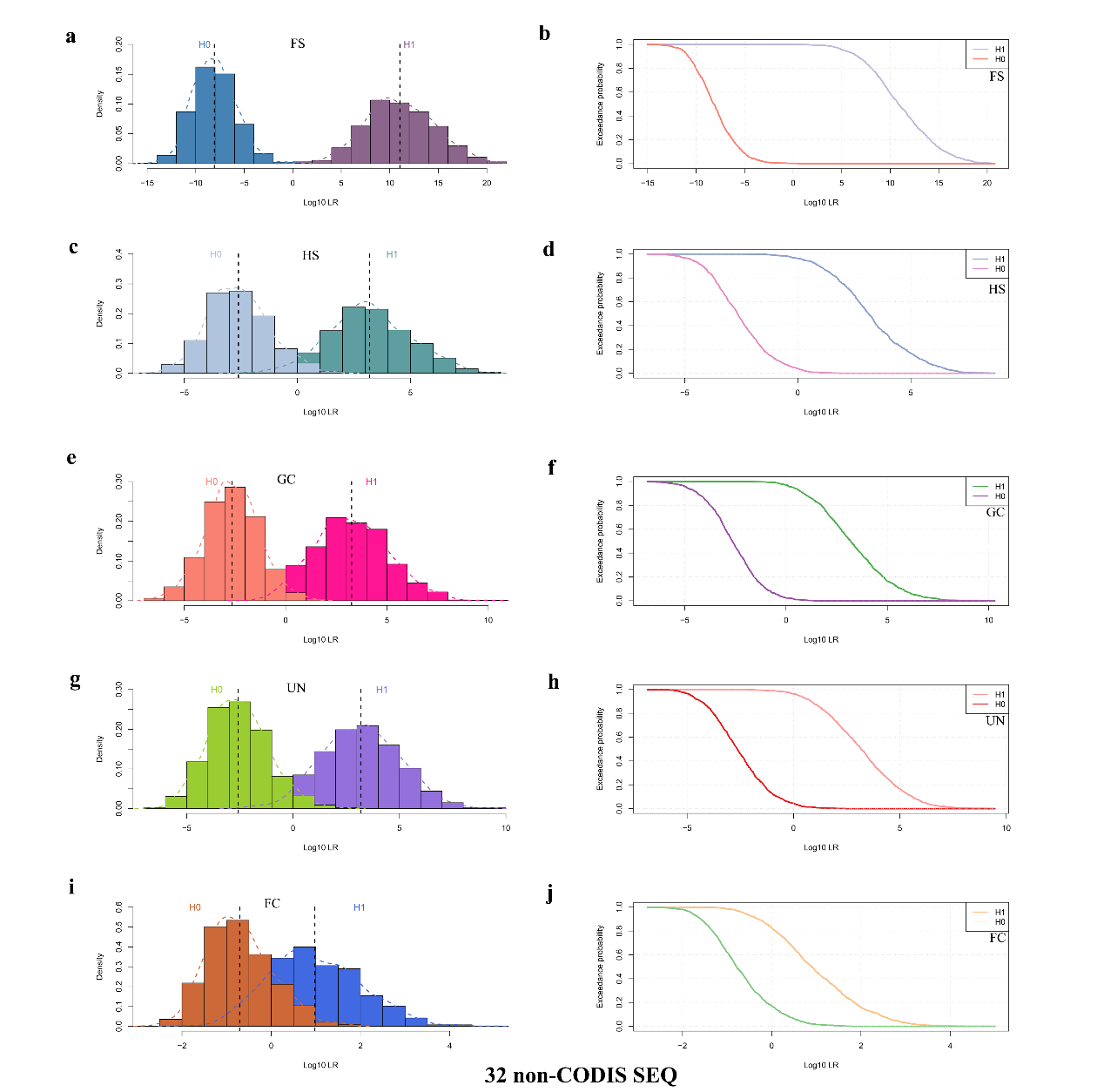


**Supplementary Fig. 4.** Frequency distribution histograms and expected probability density distribution curves of Log_10_(LR) for kinship pairs and unrelated individual pairs based on sequence polymorphisms at 32 non-CODIS STRs (32 non-CODIS SEQ) in the Baoan group. Frequency distribution histograms of Log_10_(LR) based on sequence polymorphisms at 32 non-CODIS STRs for FS pairs and unrelated individual pairs (a), for HS pairs and unrelated individual pairs (c), for GC pairs and unrelated individual pairs (e), for UN pairs and unrelated individual pairs (g), and for FC pairs and unrelated individual pairs (i). Expected probability density distribution curves of Log_10_(LR) based on sequence polymorphisms at 32 non-CODIS STRs for FS pairs and unrelated individual pairs (b), for HS pairs and unrelated individual pairs (d), for GC pairs and unrelated individual pairs (f), for UN pairs and unrelated individual pairs (h), and for FC pairs and unrelated individual pairs (j). LR, likelihood ratio; FS, full-sibling; HS, half-sibling; GC, grandparent-grandchild; UN, uncle-nephew; FC, first cousin.
